# Case report: Expanding the understanding of the adult polyglucosan body disease continuum: novel presentations, diagnostic pitfalls, and clinical pearls

**DOI:** 10.3389/fgene.2023.1282790

**Published:** 2023-12-18

**Authors:** Matthew M. Gayed, Paulo Sgobbi, Wladimir Bocca Viera De Rezende Pinto, Priya S. Kishnani, Rebecca L. Koch

**Affiliations:** ^1^ Division of Medical Genetics, Department of Pediatrics, Duke University Medical Center, Durham, NC, United States; ^2^ Division of Neuromuscular Diseases, Department of Neurology and Neurosurgery, University of São Paulo (UNIFESP), São Paulo, Brazil

**Keywords:** adult polyglucosan body disease, glycogen storage disease type IV, whole exome sequencing, patient-reported outcome, polyglucosan body neuropathy

## Abstract

**Introduction:** Adult polyglucosan body disease (APBD) has long been regarded as the adult-onset form of glycogen storage disease type IV (GSD IV) and is caused by biallelic pathogenic variants in *GBE1*. Advances in the understanding of the natural history of APBD published in recent years have led to the use of discrete descriptors (“typical” versus “atypical”) based on adherence to traditional symptomatology and homozygosity for the p.Y329S variant. Although these general descriptors are helpful in summarizing common findings and symptoms in APBD, they are inherently limited and may affect disease recognition in diverse populations.

**Methods:** This case series includes three American patients (cases 1–3) and four Brazilian patients (cases 4–7) diagnosed with APBD. Patient-reported outcome (PRO) measures were employed to evaluate pain, fatigue, and quality of life in cases 1–3.

**Results:** We describe the clinical course and diagnostic odyssey of seven cases of APBD that challenge the utility and efficacy of discrete descriptors. Cases 1–3 are compound heterozygotes that harbor the previously identified deep intronic variant in *GBE1* and presented with “typical” APBD phenotypically, despite lacking two copies of the pathogenic p.Y329S variant. Patient-reported outcome measures in these three cases revealed the moderate levels of pain and fatigue as well as an impacted quality of life. Cases 4–7 have unique genotypic profiles and emphasize the growing recognition of presentations of APBD in diverse populations with broad neurological manifestations.

**Conclusion:** Collectively, these cases underscore the understanding of APBD as a spectrum disorder existing on the GSD IV phenotypic continuum. We draw attention to the pitfalls of commonly used genetic testing methods when diagnosing APBD and highlight the utility of patient-reported outcome questionnaires in managing this disease.

## 1 Introduction

Adult polyglucosan body disease (APBD) is the adult-onset form of glycogen storage disease type IV (GSD IV) and is caused by biallelic pathogenic variants in *GBE1* which encodes the glycogen-branching enzyme (GBE) (Robitaille et al., 1980; Mochel et al., 2012; Souza et al., 2021). Reduced or deficient GBE activity disrupts normal glycogen synthesis and leads to the formation of glycogen with elongated outer chains resembling plant amylopectin (polyglucosan) (Koch et al., 2023). The polyglucosan aggregates and accumulates in various tissues, including the central nervous system, peripheral nervous system, skeletal muscle, heart, and lungs (Peress et al., 1979; Robitaille et al., 1980; Postler et al., 2002; Sindern et al., 2003; Dainese et al., 2013; Sullivan et al., 2019). Characteristic symptoms of APBD include progressive neurogenic bladder, spastic paraparesis, and sensorimotor axonal peripheral neuropathy, with the typical onset between the fourth and sixth decades of life (Robitaille et al., 1980; Mochel et al., 2012; Koch et al., 2023). While formal epidemiological studies categorizing the number of patients affected globally are yet to be conducted, it has been established that the pathogenic missense variant, NM_000158.4(*GBE1*):c.986A>C (p.Tyr329Ser; p.Y329S) (National Center for Biotechnology Information, 2023a), occurs with greater frequency in patients with Ashkenazi Jewish ancestry (estimated carrier rate 1:35–1:48) (Bao et al., 1996; Hussain et al., 2012; Schwartz et al., 2020).

APBD was first described by Robitaille et al. (1980) and Mochel et al. (2012) published the first natural history study in a cohort of 50 patients with APBD. This foundational work detailed APBD symptomatology and common misdiagnoses, as well as described the characteristic findings on magnetic resonance imaging (MRI): hyperintense white matter abnormalities predominant in the periventricular region, as well as medullary and spinal atrophy, on T2-weighted/fluid-attenuated inversion recovery (FLAIR) imaging (Mochel et al., 2012). The investigation was conducted primarily in patients homozygous for the p.Y329S variant but also included a subset of heterozygote patients without a known second variant (Mochel et al., 2012). Shortly thereafter, Akman et al. (2015) identified a novel pathogenic deep intronic variant, NM_000158.4(GBE1):c.2053-3358_2053-3350delinsTGTTTTTTACATGACAGGT (National Center for Biotechnology Information, 2023b) (herein referred to as the identified deep intronic variant), in symptomatic patients heterozygous for the p.Y329S variant without a previously known second mutation. Since the discovery of this identified deep intronic variant, there have not been extensive, longitudinal phenotypic descriptions of patients confirmed to have this intronic variant (Akman et al., 2015; Grunseich et al., 2021). Although clinical heterogeneity has been acknowledged, more recent work in 2021 sought to delineate APBD into categories of “typical” versus “atypical” on the basis of adherence to the aforementioned traditional symptomatology and homozygosity for the p.Y329S variant (Souza et al., 2021). Yet, APBD has been reported in a variety of patients of various ages, with or without Ashkenazi Jewish ancestry, and there continues to be additional phenotypes reported through multiple case series since the initial characterizations of the disorder (Ferguson et al., 1983; Bruno et al., 1993; Ziemssen et al., 2000; Klein et al., 2004; Billot et al., 2013; Souza et al., 2021).

Due to symptoms overlapping with other neurological disorders, APBD is frequently misdiagnosed; the most common misdiagnoses include multiple sclerosis, cerebral small vessel disease, peripheral neuropathy, amyotrophic lateral sclerosis, and benign prostatic hypertrophy in men (Hellmann et al., 2015). As a result, APBD patients often face prolonged diagnostic delays and may undergo unnecessary and potentially invasive procedures (Hellmann et al., 2015). Delay in diagnosing APBD is likely complicated by a number of factors including lack of awareness regarding the disorder and its associated presentations (Mochel et al., 2012; Hellmann et al., 2015). Collective understanding also continues to be hindered by a lack of longitudinal, comprehensive clinical history data in the medical literature, particularly for those with uncommon genotypes or presenting in earlier life. To address this, we present the clinical history and diagnostic odyssey for multiple patients with APBD receiving care in the United States and Brazil.

We describe three patients from the United States who are compound heterozygotes for the identified deep intronic variant in *GBE1* and utilize patient-reported outcome (PRO) measures to shed light on quality of life as well as other functional limitations: fatigue and pain. In addition, we detail four cases from Brazil with novel *GBE1* genotypes not previously described in patients with APBD and with broad neurological presentations.

## 2 Materials and methods

### 2.1 Chart review and case descriptions

As part of an international collaboration, patients with a confirmed diagnosis of APBD were included for participation in a longitudinal, retrospective natural history study (Duke University Institutional Review Board Pro00060753, ClinicalTrials.gov Identifier: NCT02683512). All available paper and electronic medical records were reviewed for participants who consented to the study. Collaborating institutions managing patients with APBD for whom direct consent was not possible submitted de-identified participant data using a targeted, study-specific spreadsheet. Participant data were entered into REDCap electronic data capture tools hosted at Duke University. Ages reported in this study represent available information in patient records and an estimation when exact ages were not available.

### 2.2 Patient-reported outcome measures

To evaluate symptoms of pain and fatigue as well as health-related quality of life, patients were asked to complete a series of validated PRO surveys. Due to institution research ethics restrictions, only United States-based participants were eligible to complete the survey (patients 1–3). The details of each validated survey and scoring are provided in Supplementary Material. Surveys were distributed and returned in April 2023. The results represent one time point (Supplementary Table S1). Survey scoring and selection details are provided in Supplementary Material.

Aspects of pain and its interference with daily life were measured using the Brief Pain Inventory (BPI) (Cleeland and Ryan, 1994; Tan et al., 2004; Güngör et al., 2013) and the Patient-Reported Outcomes Measurement Information System (PROMIS) Short Form v1.1—Pain Interference 8a (PI-8a) (Amtmann et al., 2010; Cella et al., 2010; Rothrock et al., 2010). Fatigue and its impact on quality of life were measured using the Brief Fatigue Inventory (BFI) (Mendoza et al., 1999; Shahid et al., 2012; Liu et al., 2022; Ritchie et al., 2023) and the PROMIS Short Form v1.0—Fatigue 13a (FACIT-Fatigue) (Lai et al., 2011; Cella et al., 2016). Here, pain and fatigue as measured by the BPI and BFI, respectively, were defined as no interference (0), mild (1–3), moderate (4–6), and severe (7–10) (Serlin et al., 1995; Mendoza et al., 1999; Li et al., 2007; Deandrea et al., 2008; Shahid et al., 2012; Güngör et al., 2013; Liu et al., 2022; Ritchie et al., 2023). PI-8a and FACIT-Fatigue T-scores were calculated using HealthMeasures Scoring Service, powered by Assessment Center (https://www.assessmentcenter.net/ac_scoringservice), and cut-offs for normal, mild, moderate, and severe pain interference were defined as >55, 55 to >60, 60 to >70, and >70, respectively (Cella et al., 2010; Rothrock et al., 2010; HealthMeasures, 2023).

Quality of life was measured using the EuroQol Research Foundation EQ-5D-5L (Janssen et al., 2013; Devlin et al., 2018; Janssen et al., 2018; Pickard et al., 2019) survey and the RAND Corporation 36-Item Health Survey 1.0 (SF-36) (Ware and Sherbourne, 1992; RAND Corporation, 2023). The EQ-5D-5L measures the quality of life across five dimensions: mobility, self-care, usual activities, pain/discomfort, and anxiety/depression; health states were then converted into a utility index value for each participant (Janssen et al., 2013; Devlin et al., 2018; Janssen et al., 2018; EuroQol Research Foundation, 2019; Pickard et al., 2019). The SF-36 assesses quality of life in eight domains: physical functioning, role limitations due to physical health, role limitations due to emotional problems, energy/fatigue, emotional wellbeing, social functioning, pain, and general health (Ware and Sherbourne, 1992; RAND Corporation, 2023). Individual patient utility index values (EQ-5D-5L) and health scores (SF-36) were then compared to published norm values (Jiang et al., 2021; RAND Corporation, 2023).

## 3 Results

### 3.1 Cases of APBD with the identified deep intronic variant in *GBE1* underscore the pitfalls of common genetic testing methods

#### 3.1.1 Case 1

Case 1 is a White American male with known Ashkenazi Jewish ancestry who initially presented at age 51.4 years due to fecal incontinence and urinary retention. He reported to the emergency department due to an episode of sudden, forceful loss of fecal contents. Preliminary abdominal X-ray imaging returned benign, and he was recommended to begin fiber supplementation. Of note, he was also found to be retaining urine at this visit. At age 52.6 years following multiple subsequent episodes of fecal incontinence, he was evaluated by the gastroenterology department and anorectal manometry was performed, revealing sphincter dysfunction. MRI of the lumbar spine at 52.7 years did not reveal a cause for the fecal incontinence or sphincter dysfunction. MRI of the brain without contrast, followed by MRA (magnetic resonance angiography) of the head and, subsequently, MRI of the brain with and without contrast and of the cervical, thoracic, and lumbar spine were performed together, revealing evidence of leukodystrophy with findings of white matter abnormalities, cerebral and cerebellar volume loss, spinal cord atrophy, and vascular anomalies (Table 1). Evaluation by the neurology department at age 53.3 years disclosed a history of bladder dysfunction and erectile dysfunction from age 44.5 years, as well as gait abnormalities starting at age 49.6 years. Bladder dysfunction was diagnosed as benign prostatic hypertrophy, although a review of records showed resistance to multiple medications. He was noted to have bilateral pes cavus, ataxic gait, and abnormal reflexes (increased knee jerk, decreased ankle jerk, and upgoing plantar reflex bilaterally). A leukodystrophy gene panel returned without notable findings. At age 53.5 years, he underwent cystoscopy, showing urethral obstruction (Table 2), and had a prostatic urethral lift procedure at age 54.0 years. Despite this procedure, he continued to develop symptoms of urinary retention and elevated post void residuals. To further evaluate gait disturbances, he underwent the electromyography and nerve conduction velocity test (EMG/NCS) at age 54.4 years, showing a length-dependent sensorimotor axonal neuropathy in the right lower extremity (Table 3).

**TABLE 1:** Results of MRI in cases with APBD.

Case	Age (years)	Image location	Summary of MRI findings
Case 1	52.7	Lumbar spine	Multilevel discogenic and facet hypertrophic degenerative change with mild central canal and foraminal stenosis
	53.2	Brain	Moderate diffuse cerebral volume loss and marked diffuse cerebellar volume loss. Diffuse confluent signal abnormality in the supratentorial white matter, brainstem, middle cerebellar peduncles, and bilateral cerebral hemispheres with signal abnormalities extending into the cervical spine. Prominent linear susceptibility artifact in right greater than left cerebral peduncles
	53.2	Brain MRA	Infundibulum at the origin of the left inferolateral trunk. Subtle contour irregularity of the vertical portion of the left vertebral artery at the C2 level
	53.2	Brain	Stable diffuse severe FLAIR hyperintensity throughout supratentorial and infratentorial white matter. Abnormal T2/FLAIR signals in the brainstem most pronounced at the cerebral peduncles and centrally within the medulla and pyramids. Mild supratentorial volume loss and severe cerebellar and brainstem volume loss
	53.2	Cervical spine	Small spinal cord with signal abnormalities. Disc herniation causing narrowing of the canal at C6–C7. Abnormal tortuous vasculature in dorsal paraspinal soft tissue
	53.2	Thoracic spine	Diffusely small spinal cord with abnormal signals. Hemangiomas within multiple vertebral bodies. Abnormal tortuous vasculature of the paraspinal soft tissues
	53.2	Lumbar spine	Multilevel degenerative change most prominent at L3–L4 and L4–L5. Large extraspinal synovial cysts at L3–L4 larger than those reported in prior studies
Case 2	62.0	Cervical spine	Mild cervical spondyloarthropathy at C4–C5 and C5–C6. Atrophy of the brainstem, cerebellum, and cervical cord
	62.0	Thoracic spine	Moderately severe S-shaped scoliosis and moderate generalized atrophy of the thoracic cord
	62.0	Lumbar spine	Moderate dextrorotatory scoliosis of the mid-to-upper lumbar spine. Multilevel lumbar disc desiccation and degeneration. Small L3–L4 and L5–S1 disc protrusions contributing to foraminal stenosis. Small posterior central L5–S1 disc protrusion
	63.9	Brain	Confluent T2/FLAIR hyperintensities throughout the supratentorial and infratentorial white matter, extending to the juxtacortical regions including the anterior temporal lobes. Severe atrophy of the midline cerebellum with milder cerebellar atrophy. Atrophy of the midbrain/medulla with relative sparing of the pons. Cervical spinal cord atrophy as previously demonstrated. Moderate diffuse cortical atrophy. Hemosiderin signals in the bilateral pallidum, red nucleus, and dentate nuclei
	64.7	Brain	Extensive confluent signal abnormality in bilateral cerebral white matter with volume loss. No significant change from prior studies. T1 hypointensity in bilateral parietal and occipital white matter and along the cortical spinal tracts. Moderate atrophy of cerebral and cerebellar hemispheres. Disproportionate atrophy of the brainstem and cerebellar vermis
Case 3	60.6	Brain	Severe patchy and confluent T2/FLAIR hyperintensities in the periventricular, deep, and subcortical white matter bilaterally with involvement of the pons. Parenchymal volume loss with enlargement of the ventricles greater than sulci
	60.9	Brain	Extensive signal abnormality in bilateral cerebellar hemispheres, mostly confluent, but also more focal areas of signal abnormality. Some are associated with cystic changes. The region of the cerebellar dentate nuclei is markedly abnormal. Marked atrophy of the cervical spinal cord, cerebellar vermis and tonsils, medulla, and portions of the cerebellar hemispheres
	60.9	Cervical and thoracic spine	Marked spinal cord thinning. Mild spondylosis. Lumbar scoliosis
	63.0	Pelvis	No pathology
	64.4	Right hip	Degenerative anterior superior and superior labral tear. Partial tear of the gluteus minimus tendon. Hamstring tendinosis
	64.4	Cervical, thoracic, and lumbar spine	Diffuse atrophy of the spinal cord, slightly progressive from prior studies. Severe atrophy of the medulla. Multilevel degenerative changes in the cervical spine with mild canal narrowing and right foramen at the C5–C6 level. Multilevel degenerative changes in the lumbar spine
Case 4	31.0	Brain	Small hyperintense foci in the subcortical and periventricular areas on T2/FLAIR
Case 5	50.0	Brain	Severe patchy and confluent T2/FLAIR hyperintensities in the periventricular, deep, and subcortical white matter bilaterally with involvement of the pons. Parenchymal volume loss with the enlargement of the ventricles greater than sulci
Case 6	47.0	Brain	Periventricular white matter abnormalities characterized by confluent T2/FLAIR hyperintensities throughout the supratentorial compartment with cerebellar atrophy predominantly on vermis and thin corpus callosum
Case 7	72.0	Brain and spine	Diffuse, confluent, and multifocal leukoencephalopathy with profound periventricular predominance sparing U-fibers with moderate atrophy of the brainstem with hummingbird sign and global cerebellar atrophy

MRA, magnetic resonance angiography.

**TABLE 2:** Results of bladder studies in cases with APBD.

Case	Age (years)	Procedure type	Summary of bladder study findings
Case 1	49.6	Cystoscopy and urodynamic studies	Circumferential narrowing of the bladder neck with mild visual obstruction. Bladder with trabeculation. Normal bladder capacity and compliance. Detrusor pressure relatively weak and non-sustained. Incomplete emptying
	53.5	Cystoscopy and transurethral ultrasound	Bilobar enlargement of the prostate with visible obstruction of the bladder neck. Diffuse trabeculation of the bladder with multiple wide caliber diverticula
	53.9	Cystoscopy and urodynamic studies	Moderate capacity. Uninhibited bladder contractions with urge urinary incontinence. Low-to-moderate flow and slightly low pressure
	54.3	Cystoscopy	Bladder trabeculation. No obstruction of the prostatic urethra. Improved flow following prostatic urethral lift procedure. Filling phase without uninhibited contractions and urge urinary incontinence as previously observed. Capacity improved
	54.7	Urodynamic studies	Elevated post-void residual with low Qmax
Case 3	*	Cystoscopy and urodynamic studies	Inflamed bladder with diverticula, trabeculation, and cellule formation on cystoscopy. Urodynamic evidence of overactivity, reduced capacity, and high-pressure phasic contractions
	61.9	Cystoscopy	Neurogenic trabeculated bladder with evidence of phasic instability
	65.1	CT abdomen and pelvis	Moderate bladder wall thickening and pelvic floor laxity

*Represents result at an undisclosed age prior to initial clinical evaluation.

**TABLE 3:** Results of EMG/NCS in cases with APBD.

Case	Age (years)	Summary of EMG/NCS findings
Case 1	54.4	Length-dependent motor greater than sensory axonal neuropathy with chronic neurogenic changes in the right lower extremity
	54.7	Length-dependent, axonal, sensorimotor peripheral neuropathy
Case 2	62.2	Moderately severe, predominantly axonal, sensorimotor polyneuropathy without evidence of lumbosacral radiculopathy
	64.7	Severe axonal neuropathy in the legs with chronic neurogenic changes in the needle EMG to the proximal and distal leg muscles. Carpal tunnel in the right hand as well as mild ulnar sensory neuropathy
Case 3	*	Generalized axonal sensorimotor polyneuropathy with L4–5 radiculopathy
	61.2	Motor-predominant axonal polyneuropathy vs. radiculopathy. Abnormal conduction in descending corticospinal tracts
Case 4	31.0	Length-dependent motor and sensory axonal neuropathy with chronic neurogenic changes involving the lower limbs
Case 5	50.0	Length-dependent, moderate axonal sensorimotor peripheral neuropathy involving the lower and upper limbs
Case 6	47.0	Length-dependent, mild axonal sensorimotor peripheral neuropathy with motor predominance involving the lower and upper limbs
Case 7	71.0	Length-dependent, mild axonal sensorimotor peripheral neuropathy with sensory predominance involving the lower and upper limbs

*Represents result at an undisclosed age prior to initial clinical evaluation available for review.

At age 54.6 years, he was formally diagnosed with APBD after research reanalysis of the prior leukodystrophy panel identified NM_000158.4(GBE1):c.760A>G (p.Thr254Ala; p.T254A)—a variant with conflicting interpretations of pathogenicity (National Center for Biotechnology Information, 2023d)—and the identified deep intronic variant. At age 54.7 years, he underwent repeat EMG, confirming length-dependent, axonal, sensorimotor peripheral neuropathy (Table 3) and, subsequently, began clean intermittent catheterizations due to urinary retention. He was evaluated at the Duke Metabolic Genetics clinic at age 55.5 years and reported ongoing symptoms of bladder dysfunction and gait instability, as well as intermittent episodes of fecal incontinence at least once every 6 months. During that visit, he reported an overall improvement of bladder control with clean intermittent catheterizations and has not had any resultant infections. However, he disclosed experiencing increased fatigue, noting that he was no longer able to walk distances greater than a mile due to sensation of muscle weakness as well as profound tiredness following meals. Muscle weakness was further evaluated using muscle ultrasound, with findings suggestive of myopathy in conjunction with an elevated creatine kinase level (516 U/L). Case 1 has no known liver or cardiac manifestations of GSD IV; routine echocardiograms and electrocardiograms performed between ages 47.6 and 54.7 years were normal, and alanine transaminase (ALT) levels have always been within normal limits, most recently measured at age 56.0 years.

#### 3.1.2 Case 2

Case 2 is a White American female with Ashkenazi Jewish ancestry, who presented at age 61.9 years to an orthopedist with symptoms of worsening gait, frequent falls, poor hand dexterity, and paresthesia and pain in the proximal left lower extremity initially thought to be due to worsening of longstanding kyphoscoliosis. On further review, postural changes and gait instability began as early as ages 45 and 55 years, respectively. MRI of the spine at age 62.0 years revealed atrophy of the cerebellum, cervical, and thoracic spinal cord (Table 1). EMG/NCS performed at age 62.2 years also displayed evidence of polyneuropathy (Table 3).

At age 62.7 years, she was evaluated by the neurology department, at which time she endorsed muscle tightness/stiffness as well as cramping of the right hand, rollator use from approximately age 60 years, as well as long-standing bladder dysfunction from as early as age 49 years beginning initially as urgency and progressing to incontinence requiring the use of pads. An ataxia gene panel and subsequent whole exome sequencing on the same sample were performed at age 63.6 years and returned negative; notably, *GBE1* analysis was not performed on the ataxia panel nor was it commented on in the whole exome sequencing report. Following undiagnostic genetic testing results, MRI of the brain was performed at age 63.9 years, revealing white matter hyperintensities as well as cortical, midbrain, and midline cerebellar atrophy (Table 1). At age 64.0 years, mitochondrial gene testing and targeted *FMR1* testing were performed and returned negative. She had worsening symptoms including deteriorating balance, weakness of the right leg, and frequent falls. On examination, she was noted to have abnormal reflexes, abnormal gait, and paratonia in the lower extremities. At subsequent evaluations, she reported increased dependency on a rollator for mobility and increasing difficulties with daily life activities.

She was referred for a neurological specialty evaluation at age 64.7 years at which point she reported the continuing progression of symptoms including lower extremity stiffness and weakness as well as bilateral foot drop and progression of bladder dysfunction, with excessive straining to urinate. EMG/NCS indicated severe axonal neuropathy in the lower extremities, in addition to incidental carpal tunnel syndrome in the right arm. Repeat MRI of the brain again showed extensive white matter signal abnormalities as well as cerebral, brainstem, and cerebellar atrophy. Reanalysis of molecular testing was performed at age 65.2 years, detecting the p.Y329S and identified deep intronic variants in *GBE1*.

Case 2 was reported to have a history of anxiety and was subjectively reported to have declining short-term memory throughout the diagnostic work up period without formal cognition evaluations. She has no known cardiac or hepatic manifestations of GSD IV; ALT levels most recently measured at age 64.7 years were within normal limits.

#### 3.1.3 Case 3

Case 3 is a White American female with known Ashkenazi Jewish ancestry who initially presented to a specialty multiple sclerosis clinic at age 60.9 years with bladder and bowel dysfunction and increasing difficulty with balance and gait. Bladder dysfunction manifested at approximately age 51 years as increased urgency and later progressed to incontinence and extensive urinary tract infections. Gait instability also began at approximately age 51 years with worsening of coordination and balance over time. She also reported a history of bowel dysfunction marked by constipation and fecal incontinence. Prior to initial evaluation, peripheral neuropathy was noted on EMG/NCS and extensive non-enhancing leukodystrophy on brain MRI (Table 1). Urodynamic studies and cystoscopy revealed reduced bladder capacity and overactivity in addition to bladder wall trabeculation and cellule formation (Table 2).

She was reevaluated at age 61.2 years for adult-onset leukodystrophy. At this visit, she further reported significant fatigue with the onset as early as age 59 years accompanied by frequent headaches and neck pain. She also noted the limited intermittent use of a wheelchair due to gait instability. Physical examination revealed diminished reflexes accompanied by a positive Babinski sign and an unsteady, wide-based gait. Repeat EMG/NCS disclosed prolonged central motor conduction times, indicating abnormal conduction in the descending corticospinal tracts in addition to a motor-predominant axonal polyneuropathy versus polyradiculopathy (Table 3). Mitochondrial DNA testing and whole exome sequencing were performed, and both returned as non-diagnostic. Because of the clinical suspicion for APBD, further evaluation and subsequent gene sequencing of the *GBE1* gene led to a diagnosis of APBD at age 62.1 years. She had two pathogenic *GBE1* variants (p.Y329S and the identified deep intronic variant) as well as GBE activity less than 10% compared to normal in skin fibroblasts.

Case 3 experienced ongoing deterioration since the time of diagnosis characterized by worsening bladder dysfunction, pain, gait instability, and cognition. Repeat cystoscopy at age 61.9 years again demonstrated neurogenic bladder dysfunction (Table 2). A trial of desmopressin (DDAVP) and pelvic floor physical therapy did not alleviate the symptoms. At age 65.1 years, a CT scan of the abdomen and pelvis revealed moderate bladder wall thickening and pelvic floor laxity/cystocele. She continues to have difficulty with urinary incontinence, voiding, urinary stream strength, recurrent UTIs, and perineal irritation.

Case 3 reports a significant history of pain throughout her disease course. At age 62.1 years, she began experiencing neuropathic, burning pain in the lower extremities up to the level of the thighs. Bladder/pelvic/perineal pain was reported as early as 62.8 years. Due to the unremitting, disabling nature of her pain, case 3 has trialed a number of therapies, such as a vibration table, duloxetine, transcranial magnetic stimulation, spinal procedures (selective nerve root blocks and pulsed radiofrequency ablations), and scrambler therapy—none of which alleviated her pain.

She has noted worsening gait instability and progressed from using a cane to a walker at age 64.3 years and then an increased reliance on a wheelchair by age 64.9 years. Despite regular physical therapy, her condition continues to deteriorate. Case 3 has no known cardiac or hepatic manifestations of GSD IV; she had a normal abdominal CT scan at age 65.1 years, and ALT levels most recently measured at 61.2 years old were within normal limits. Notably, she has a history of acute adjustment disorder with anxiety and insomnia.

### 3.2 PRO measures: pain, fatigue, and quality of life

PROs were used to further evaluate the health statuses for cases 1–3 across a series of domains including fatigue, pain, and quality of life (Table 4). On the BPI, averaged scores for pain severity and pain interference were 5.5 (moderate) and 6.1 (moderate), respectively. The mean T-score for the PI-8a was 62.7 (moderate pain interference). On the BFI, the average global fatigue score was 6.5 (moderate); the greatest impacted domain on average was walking ability. The mean T-score for the FACIT-Fatigue was 60.0 (moderate fatigue). Average scores for quality of life domains as measured by the SF-36 were lower than the established national averages, reflecting a significant impact on quality of life. Lastly, EQ-5D-5L assessed quality of life, and the health profiles were 32,233 (case 1), 54,532 (case 2), and 42,442 (case 3); these equate to utility index values of 0.5, 0.048, and 0.044, respectively. When compared to the US national mean utility index value (mean = 0.851, SD = 0.205), case 1 has an index value falling to approximately 1.7 SD from the mean, and cases 2 and 3 have index values falling to 3.9 SD from the mean.

**TABLE 4:** Results of PRO measures from patients with APBD.

Pain		
**BPI**	**Average value (n = 3)**	
Pain severity	5.5 (3.5–7)	
Pain interference	6.1 (4.0–8.7)	
**PI-8a**	**Average value (n = 3)**	Standard deviation*
T-score	62.7 (55.9–70.7)	1.3

**Fatigue**		
**BFI**	**Average value (n = 3)**	
Current fatigue	6.3	
Usual fatigue	6.7	
Worst fatigue	8.7	
General activity	6.3	
Mood	5.7	
Walking ability	8.0	
Normal work	6.7	
Relations with others	4.7	
Enjoyment	5.7	
Global fatigue score	6.5	
**FACIT-Fatigue**	**Average value (n = 3)**	Standard deviation*
T-score	60.0 (55.3–66.5)	1.0

**Quality of life**		
**SF-36**	**Average value (n = 3)**	Standard deviation*
Physical functioning	23.3	1.7
Role functioning/physical	25.0	0.7
Role functioning/emotional	55.6	0.3
Energy/fatigue	20.0	1.4
Emotional wellbeing	56.0	0.7
Social functioning	25.0	2.1
Pain	40.8	1.2
General health	21.7	1.7
**EQ-5D-5L**	**Utility index**	Standard deviation*
Patient 1	0.5	1.7
Patient 2	0.048	3.9
Patient 3	0.044	3.9

*Represents standard deviations from the population mean value.

Validated PRO measures assessed pain, fatigue, and quality of life in patients 1–3. Pain and its interference with daily life were assessed using Brief Pain Inventory (BPI) and the Patient-Reported Outcomes Measurement Information System (PROMIS) Short Form v1.1—Pain Interference 8a (PI-8a). The most common pain descriptors on BPI were aching, gnawing, exhausting, tiring, penetrating, nagging, and miserable. Fatigue and its impact on quality of life were measured using the Brief Fatigue Inventory (BFI) and the PROMIS Short Form v1.0—Fatigue 13a (FACIT-Fatigue). Quality of life was measured using the EuroQol Research Foundation EQ-5D-5L survey and the RAND 36-Item Health Survey 1.0 (SF-36). Scores reported are averaged, and ranges are provided within parentheses for pain, fatigue, and SF-36.

### 3.3 Expanding our understanding of the APBD continuum: novel genotypes and earlier clinical presentation

#### 3.3.1 Case 4

Case 4 is a White Brazilian female with no known Ashkenazi Jewish ancestry. She presented with urinary urgency beginning at age 26 years, followed by frequent urinary tract infections at age 28 years. Bladder dysfunction was notable for urgency and difficulty in emptying. At age 30 years, she experienced muscle cramping in the lower extremities (peroneal muscle group bilaterally) and small-fiber neuropathy symptoms in the distal lower extremities characterized by sensations of burning and tingling. At age 31 years, she exhibited muscle wasting, muscle weakness, easy fatiguability, and pain in the lower limb muscle groups as well as peripheral neuropathy characterized by hyperesthesia and paresthesia. At this time, NCS was performed, which disclosed sensorimotor axonal neuropathy (Table 3). Brain imaging revealed small foci of hyperintensity in the subcortical and periventricular areas on FLAIR and T2-weighted imaging (Table 1). At age 31 years, she underwent gene panel testing for inherited peripheral neuropathies which revealed homozygosity for NM_000158.4(GBE1):c.292G>C (p.Val98Leu; p.V98L), interpreted as a variant of uncertain significance (VUS) in ClinVar (National Center for Biotechnology Information, 2023e). She was not reported to have an unsteady or spastic gait or any cognitive deficits as of age 31 years.

#### 3.3.2 Case 5

Case 5 is a White Brazilian male with no known Ashkenazi ancestry presenting with numbness and paresthesia in the lower limbs. At age 30, he reported experiencing symptoms of small-fiber neuropathy characterized by burning and tingling in the distal lower extremities. Between age 30 and 50 years, he was misdiagnosed and symptomatically treated for sporadic and idiopathic small-fiber neuropathy with pregabalin without resolution. Evaluation at age 50 years also reported diffuse muscle pain involving the quadriceps femoris muscles and the gastrocnemii bilaterally not associated with exercise. He also had symptoms of peripheral neuropathy, and NCS confirmed moderate axonal sensorimotor polyneuropathy involving the lower and upper limbs (Table 3). He began experiencing bladder dysfunction at age 50 years marked by increased urgency. Physical examination revealed decreased strength in hip extensors, hip flexors, and ankle plantar flexion. Brain MRI revealed periventricular white matter hyperintensities and parenchymal volume loss (Table 1). Genetic testing with a multigene panel for inherited neuropathies at age 50 years revealed homozygosity for NM_000158.4(GBE1):c.534G>C (p.Trp178Cys; p.W178C), interpreted as a VUS (National Center for Biotechnology Information, 2023f).

#### 3.3.3 Case 6

Case 6 is a White Brazilian female with no known Ashkenazi ancestry who presented with bradykinesia and spastic paraparesis since age 35 years. There was an initial clinical suspicion for juvenile monogenic Parkinson’s disease or neurotransmitter disorders such as Segawa syndrome; a further review of history revealed good clinical improvement with low doses of levodopa. Previous medical history detailed bladder dysfunction beginning at age 32 years, including urinary incontinence and frequent urinary tract infections, as well as constipation beginning at age 44 years. She began using a cane at age 45 years and reported frequent daily falls and continuing unsteady gait on evaluation at age 47 years. NCS performed at 45 years revealed mild axonal sensorimotor polyneuropathy involving the upper and lower limbs (Table 3). At age 47 years, brain MRI identified cerebellar atrophy with periventricular white matter abnormalities (Table 1), and gene panel testing for neurodegenerative disorders identified p.V98L and NM_000158.4(GBE1):c.1909C>T (p.Arg637Ter; p.R637*), the latter interpreted as pathogenic/likely pathogenic (National Center for Biotechnology Information, 2023c). Physical examination from the time of diagnosis revealed preserved muscle strength and normal deep tendon reflexes with bradykinesia, mild spasticity in the lower limbs, and postural instability as well as cognitive impairment [Montreal Cognitive Assessment (MoCA): 22/30] and major depressive disorder.

#### 3.3.4 Case 7

Case 7 is a Black Brazilian male with no known Ashkenazi Jewish ancestry presenting with constipation, bladder dysfunction, erectile dysfunction, muscle cramping, spasticity, and unsteady gait. Of note, family history is positive for consanguinity as parents are first-degree cousins. The onset of constipation occurred at age 60 years. Bladder dysfunction was characterized by incontinence accompanied by frequent urinary tract infections starting at age 65 years, and EMG/NCS at age 71 years confirmed axonal sensorimotor polyneuropathy (Table 3). At age 72 years, physical examination revealed overall brisk tendon reflexes and reduced strength in hip flexion and extension bilaterally. Brain and spine MRI at age 72 years revealed evidence of leukoencephalopathy with periventricular predominance and atrophy of the brainstem and cerebellum (Table 1). A gene panel for neurodegenerative disorders at age 72 years revealed homozygosity for NM_000158.4(GBE1):c.929A>C (p.Tyr310Ser; p.Y310S). This variant has not been previously reported in the literature in an individual with APBD or entered in ClinVar, nor is it present in the population database gnomAD. Currently, there is limited information on this variant, and thus it is classified as a VUS. As of age 73 years, he began experiencing frequent falls and used a walker for mobility. A review of medical records further revealed history of misdiagnosis with Parkinson’s disease and normal pressure hydrocephalus.

## 4 Discussion

Through our work, we present the detailed, longitudinal clinical history of seven patients with APBD, with the goal of expanding the collective understanding of the phenotypic presentation of APBD.

We began by describing in detail the clinical course of three cases confirmed to have the identified deep intronic variant in *GBE1*, for which longitudinal descriptions in the medical literature have thus far been limited (Akman et al., 2015; Grunseich et al., 2021). The clinical course and phenotypic presentation of these three cases followed the well-documented pattern of APBD progression previously established largely in populations homozygous for the *GBE1* p.Y329S variant (Mochel et al., 2012; Hellmann et al., 2015). Case 1 is the first known case to be reported harboring the identified deep intronic variant without concomitantly harboring the p.Y329S variant in *GBE1*. This patient presented with bladder dysfunction at age 44 years, followed by gait abnormalities at 49 years, fecal incontinence at 54 years, and progressive fatigue at 55 years. Case 2 developed postural changes at age 45 years, followed by bladder dysfunction at age 49 years, gait instability at 55 years, and peripheral neuropathy at 62 years. Case 3 presented with bladder and bowel dysfunction as well as gait instability at age 51 years; these symptoms were followed by pain resistant to treatment, affecting various parts of the body including the neck, back, legs, and perineal region in the groin. Previous work has suggested defining cases of APBD as “typical” versus “atypical,” largely based on genotype by grouping “typical” cases with homozygosity for the p.Y329S variant in *GBE1* (Souza et al., 2021). Cases 1–3 described here displayed features associated with “typical” APBD, despite not harboring homozygosity for the p.Y329S variant. Based on this and other cases in the literature, we suggest avoiding the use of these dichotomous descriptors and instead recommend using the term “APBD” to describe all patients with this diagnosis regardless of genotypic/phenotypic presentation.

We then presented three patients that expanded our understanding of the phenotypical presentation of APBD. Clinically, cases 4–6 represent individuals with APBD in Brazil who are not of Ashkenazi Jewish descent and presented with “traditional” symptoms in early adulthood. Case 4 presented with bladder dysfunction starting at age 26 years, followed by neuropathy and muscle cramping at age 30 years. Case 5 presented with symptoms of small-fiber neuropathy at age 30 years and was diagnosed with APBD at age 50 years, by which time he was also experiencing muscle pain and bladder dysfunction. Case 6 reported bladder dysfunction at age 32 years, gait difficulties marked by bradykinesia and spastic paraparesis at age 35 years, and bowel dysfunction at age 44 years. These cases add to the growing body of evidence of APBD in younger, diverse populations, suggesting that greater attention should be paid to patients of all ages and demographics (Ferguson et al., 1983; Bruno et al., 1993; Ziemssen et al., 2000; Klein et al., 2004; Billot et al., 2013; Souza et al., 2021). We encourage clinicians to maintain high clinical suspicion and to not rule out an APBD diagnosis solely based on ancestry or age of onset. With continued use and improvements in whole exome sequencing and whole genome sequencing technologies, we expect that the diagnosis of GSD IV, including APBD, and our understanding of its natural history will improve.

All phenotypes on the GSD IV continuum, including APBD, are caused by deficiency in functional GBE activity. The genotypic profiles of cases 4–7, when evaluated in combination with their clinical presentation, extend the insight that APBD exists on the GSD IV clinical spectrum. Cases 4 and 6 harbored p.R637* and p.V98L in *GBE1*, which have previously been reported in patients with pediatric-onset GSD IV presenting with hepatic and/or neuromuscular involvement (Bruno et al., 2004; Janecke et al., 2004; Bruno et al., 2007; Li et al., 2010; Beyzaei et al., 2021). The p.V98L variant was previously reported in ClinVar in a patient with APBD, but no phenotypic information was available. Case 5 presented as homozygous for c.534G>C (p.W178C), a variant that has been reported in ClinVar in a patient with GSD IV but for which no phenotypic presentations have been published. The c.929A>C (p.Y310S) variant detected in case 7 was not previously reported in ClinVar or in the published literature and, therefore, was considered to be novel. Recent work has sought to establish that GSD IV symptomatology and presentation exist on a continuum with overlapping and multisystem involvement, rather than previously defined discrete hepatic or neuromuscular categories (Kiely et al., 2022). Our cases show that recognizing the presence of these variants in combination with the reported symptoms is important for understanding how APBD exists on the GSD IV continuum and for future phenotype–genotype correlations to improve genetic and prognostic counseling. Future work should focus on elucidating the multifaceted symptomatology of GBE deficiency, which has implications on management plans including long-term follow up and counseling.

In presenting a series of patients with APBD representing diverse phenotypic and genotypic presentations, we further suggest the broader inclusion of *GBE1* analysis on relevant gene testing panels. The diagnostic pathways for cases 1–3 provide particularly unique insights into the pitfalls of common genetic testing methods. Case 1 underwent genetic testing with a targeted leukodystrophy gene panel which initially returned negative. After strong clinical suspicion, subsequent research re-evaluation of the data from the test nearly 1.3 years later confirmed the diagnosis of APBD. Similarly, case 2 faced a prolonged time for diagnosis after initially receiving negative results on an ataxia gene panel and whole exome sequencing. Moreover, whole exome sequencing also proved undiagnostic for case 3; yet, because of strong clinical suspicion, her providers pursued GBE activity testing and targeted gene re-evaluation that led to her diagnosis. These patients frequently underwent numerous diagnostic studies and evaluations before diagnosis, resulting in increased patient burden and overall costs to healthcare systems. Although we continue to believe that genetic testing is the gold standard, first-line option for diagnosing patients with APBD, our cases demonstrate the common limitations and pitfalls of these methods. To this regard, with the broad phenotypic, genotypic, and radiographic presentation of APBD becoming increasingly well-characterized (Mochel et al., 2012; De Winter et al., 2022), the inclusion of *GBE1* testing on relevant gene panels, including those for leukodystrophy, ataxia, and peripheral neuropathy, is critical. For those that undergo whole exome sequencing, the limitations of this method have previously been described (Mori et al., 2017; Burdick et al., 2020) and may result in a missed diagnosis—as in cases 1–3. Therefore, until the current pitfalls with whole exome sequencing are resolved, we argue that clinicians should not be completely reliant on negative results from whole exome sequencing. Additionally, the utility of measuring GBE activity testing should not be understated. GBE activity testing can help diagnose APBD and can be conducted using cultured skin fibroblasts, skeletal muscle, leukocytes, and other tissues; diagnosis guidelines previously published include considerations when interpreting GBE activity in different tissues and cells (Koch et al., 2023). For those that are found to have decreased GBE activity and/or be a carrier of a pathogenic *GBE1* variant, re-evaluation of genetic data and targeted follow-up testing with the intent of assessing for the presence of commonly missed variants, including the identified deep intronic variant (Koch et al., 2023), is warranted.

Lastly, in light of the increasing number of therapeutic options in development for the treatment of APBD (Kakhlon et al., 2013; Kakhlon et al., 2018; Gumusgoz et al., 2021; Gentry et al., 2022; Gumusgoz et al., 2022) and recently published consensus guidelines (Koch et al., 2023), we piloted the use of PRO surveys to evaluate the domains of pain, fatigue, and quality of life in United States-based patients (cases 1–3). As a conglomerate, the surveys disclosed impairment in all assessed domains. Averaged scores revealed a moderate level of pain severity and pain interference on the BPI; these results were mirrored by responses on the PI-8a, which also indicated a moderate level of interference caused by pain on daily functioning. Regarding fatigue, averaged scores showed moderate levels of fatigue on both the BFI as well as the FACIT-Fatigue compared to the general population. Regarding quality of life, averaged results on the SF-36 showed lower quality of life scores in every domain assessed compared to the general population. The EQ-5D-5L again identified significant impairments in quality of life, with cases 1–3 harboring health index scores ranging from 1.7 to 3.9 and standard deviations lower than population normative values. While the PROs provided useful insights into functional impairment in aggregate, they showed perhaps a greater value at the individual level. Upon review of available medical records, pain was poorly characterized for cases 1 and 2. However, the use of PROs better elucidated this symptom. Similarly, fatigue was a poorly characterized symptom throughout the medical records for all surveyed patients, but the dual use of fatigue-related PROs revealed impairment. To the best of our knowledge, this is the first published implementation of PROs in patients with APBD. Given our limited sample size, our implementation of PROs was intended to be an initial proof of concept to establish use within this patient population, providing preliminary evidence to support the United States Food and Drug Administration guidance regarding the use of PROs to support clinical trials (Food and Drug Administration, 2020). Longitudinal data should be collected and analyzed to further evaluate the true utility of PROs in this population more broadly. In the future, we strongly encourage the use of PROs both in the setting of clinic visits as well the clinical trial space. These metrics provide an opportunity for an enhanced personalized medicine approach for symptom management and can serve as trackable outcomes to monitor therapeutic interventions.

The interdisciplinary, multicentered, retrospective nature of this study inherently has limitations. Over the last decade, MRI of the brain and spinal cord has proven essential in the evaluation and eventual diagnosis of individuals with APBD. Characteristic imaging findings were first described in 2012 (Mochel et al., 2012) and recently expanded on in 2022 (De Winter et al., 2022). While we provided herein the radiological findings reported by the various institutions at which our cases received care, we were unable to independently evaluate primary imaging. Future work should prospectively investigate imaging findings and symptomatology in individuals with APBD from a wide variety of genotypic backgrounds. Moreover, our ability to assess systemic manifestations of disease in our cohort was limited; our cases underwent limited to no evaluation for hepatic or cardiac involvement. Future work should aim to provide a more dedicated analysis of systemic manifestations in APBD. Lastly, the presented data on PROs represent one time point and serve as initial evidence for PRO use in this population while also highlighting their utility in understanding individual disease burden. Longitudinal data across multiple time points will prove essential in the validation of these surveys for APBD.

## 5 Conclusion

We describe in detail the clinical course of three compound heterozygotes harboring the identified deep intronic variant in *GBE1* as well as four cases from Brazil with unique genotypic profiles. We emphasize the growing presentation of APBD in diverse populations, requiring heightened awareness to ensure proper diagnosis and intervention, and suggest discontinuing the use of discrete classification categories. Lastly, we present the proof-of-concept use of PRO questionnaires to further describe the clinical states of APBD.

## Data Availability

The datasets for this article are not publicly available due to concerns regarding participant/patient anonymity. Requests to access the datasets should be directed to the corresponding author.
