# Supplementary material for: Case report: Expanding the understanding of the adult polyglucosan body disease continuum: novel presentations, diagnostic pitfalls, and clinical pearls
Source: Front Genet. 2023 Dec 18;14:1282790. doi: 10.3389/fgene.2023.1282790 (PMC10758020; doi:10.3389/fgene.2023.1282790)
Supplement: Supplementary file 1 [file Table1.DOCX]

Supplementary Material

# Patient Reported Outcome Measures

## Pain

Aspects of pain and its interference on daily life were measured using the Brief Pain Inventory (BPI) and the Patient Reported Outcomes Measurement Information System (PROMIS) Short Form v1.1 – Pain Interference 8a (PI-8a). The BPI is a validated measure used to assess pain originally developed for use in cancer related pain, and later used in a wide variety of diseases including Pompe disease, multiple sclerosis, neuropathic pain, and fibromyalgia (Cleeland and Ryan, 1994; Tan et al., 2004; Cleeland, 2009; Güngör et al., 2013). The long form of the BPI was administered to patients after permission for use was obtained. Answers were scored on two domains, pain interference and pain severity, as outlined in The BPI User Guide distributed by survey developers (Cleeland, 2009). Variations in ratings of pain have previously been used and reported (Serlin et al., 1995; Li et al., 2007; Deandrea et al., 2008; Güngör et al., 2013). Pain was defined here as no interference (0), mild (1-3), moderate (4-6), and severe (7-10). The PI-8a is validated patient reported metric to assess the impact of pain on various aspects of an individual’s life (Amtmann et al., 2010). The PI-8a was scored using the HealthMeasures Scoring Service, powered by Assessment Center (https://www.assessmentcenter.net/ac_scoringservice), which reported raw scores and t-scores for each respondent assuming a population statistics (mean = 50, SD = 10) with the general population used as the centering sample and the general population and a clinical sample used as the calibration sample (Amtmann et al., 2010; Cella et al., 2010; Rothrock et al., 2010). Patient t-scores were averaged and standard deviations away from population mean were obtained by subtracting the population mean from the patient average t-score and dividing by the standard deviation of 10. T-score cut offs for normal, mild, moderate, and severe pain interference are defined as less than 55, 55 to less than 60, 60 to less than 70, and 70 and greater (HealthMeasures; Cella et al., 2010; Rothrock et al., 2010).

## Fatigue

Fatigue and its impact on quality of life were measured using the Brief Fatigue Inventory (BFI) and the PROMIS Short Form v1.0 – Fatigue 13a (FACIT-Fatigue). The BFI is a validated metric initially used to measure fatigue in cancer patients, but has also been used in a variety of different conditions (Mendoza et al., 1999; Shahid et al., 2012; Liu et al., 2022; Ritchie et al., 2023). BFI was administered to patients and permission for use was obtained prior to distribution. BFI global fatigue score was calculated by averaging values across all nine domains assessed (Mendoza et al., 1999; Shahid et al., 2012). Scores were subsequently averaged across patients. Average domain scores are also reported. Fatigue is categorized as no fatigue (0), mild (1-3), moderate (4-6), and severe (7-10) (Mendoza et al., 1999; Shahid et al., 2012; Liu et al., 2022; Ritchie et al., 2023). The FACIT – Fatigue is a metric used to assess generalized fatigue and has utility in multiple conditions (Lai et al., 2011; Cella et al., 2016). The FACIT- Fatigue was scored using the HealthMeasures Scoring Service, powered by Assessment Center (https://www.assessmentcenter.net/ac_scoringservice), which reported raw scores and t-scores for each respondent assuming a population statistics (mean = 50, SD = 10) with the general population used as the centering and calibration sample (Cella et al., 2010; Rothrock et al., 2010; Cella et al., 2016). Patient t-scores were averaged and standard deviations away from population mean were obtained by subtracting the population mean from the patient average t-score and dividing by the standard deviation of 10. T-score cut offs for normal, mild, moderate, and severe pain interference are defined as less than 55, 55 to less than 60, 60 to less than 70, and 70 and greater (HealthMeasures; Cella et al., 2010; Rothrock et al., 2010).

## Quality of Life

Quality of life was measured using the EuroQol Research Foundation EQ-5D-5L survey and the RAND 36 Item Health Survey 1.0 (SF-36). The EQ-5D-5L is a health-related quality of life metric used around the world and validated for use in the United States population (Janssen et al., 2013; Devlin et al., 2018; Janssen et al., 2018; Pickard et al., 2019). It has also been used in glycogen storage disorders types 1a and 2 (Hubig et al.; Kanters et al., 2011; Ghajarzadeh et al., 2018; Attal et al., 2021; Mawla et al., 2021; Kruger et al., 2023). The EQ-5D-5L measures quality of life across five dimensions: mobility, self-care, usual activities, pain/discomfort, and anxiety/depression. After permission was received from EuroQol Research Foundation, surveys were distributed to patients. Patient responses were mapped to specific health states as described in the EQ-5D-5L User Guide; health states were then converted into a utility index value using a standard validated value set for the United States that attributing weights to each dimension (EQ-5D, 2019; Pickard et al., 2019). Individual patient utility index values were then compared to published norm values (Jiang et al., 2021). The SF-36 is a widely used health related QoL measure initially developed in 1992 (RAND Corporation; Ware and Sherbourne, 1992; Hays et al., 1993; VanderZee et al., 1996). The RAND corporation has made SF-36 version 1 available for open access use. The SF-36 assesses QoL in the following eight domains: Physical functioning, role limitations due to physical health, role limitations due to emotional problems, energy/fatigue, emotional well-being, social functioning, pain, and general health. Patient responses were scored as recommended by the RAND corporation; scores were generated for all 8 domains described (RAND Corporation). Scores are assigned such that a score of 0 corresponds to the lowest possible health status and a score of 100 corresponds to the best possible health status (RAND Corporation). Patient scores were averaged and compared to population norms provided by the RAND corporation (RAND Corporation; Jiang et al., 2021).

# Supplementary Table 1

PROs were used to assess individual health states and results for each patient are displayed in addition to averaged values where appropriate. *Represents standard deviations away from population mean or parameter

| **Pain** | | | | |
| --- | --- | --- | --- | --- |
| **Brief Pain Inventory** | **Patient 1** | **Patient 2** | **Patient 3** | **Average Value (n=3)** |
| Pain severity score | 3.5 | 6 | 7 | 5.5 |
| Pain interference score | 4.0 | 5.7 | 8.7 | 6.1 |
| ***Pain descriptors*** |  |  |  | ***No. of Patients*** |
| Aching | Yes | No | Yes | 2 |
| Throbbing | No | No | Yes | 1 |
| Shooting | No | No | No | 0 |
| Stabbing | No | No | Yes | 1 |
| Gnawing | Yes | No | Yes | 2 |
| Sharp | No | No | Yes | 1 |
| Tender | No | Yes | No | 1 |
| Burning | No | No | Yes | 1 |
| Exhausting | Yes | No | Yes | 2 |
| Tiring | Yes | No | Yes | 2 |
| Penetrating | Yes | No | Yes | 2 |
| Nagging | Yes | No | Yes | 2 |
| Numb | No | No | Yes | 1 |
| Miserable | No | Yes | Yes | 2 |
| Unbearable | No | No | Yes | 1 |
| **PROMIS Pain Interference - SF 8a** | **Patient 1** | **Patient 2** | **Patient 3** | **Average Value (n=3)** |
| Raw score | 24 | 16 | 37 |  |
| t-score | 61.4 | 55.9 | 70.7 | 62.7 |
| Standard deviation* |  |  |  | 1.3 |
| **Fatigue** | | | | |
| **Brief Fatigue Inventory** | **Patient 1** | **Patient 2** | **Patient 3** | **Average Value (n=3)** |
| Current fatigue | 3 | 8 | 8 | 6.3 |
| Usual fatigue | 5 | 7 | 8 | 6.7 |
| Worst fatigue | 8 | 9 | 9 | 8.7 |
| General activity | 3 | 7 | 9 | 6.3 |
| Mood | 3 | 5 | 9 | 5.7 |
| Walking ability | 5 | 10 | 9 | 8.0 |
| Normal work | 2 | 8 | 10 | 6.7 |
| Relations with others | 3 | 3 | 8 | 4.7 |
| Enjoyment | 5 | 5 | 7 | 5.7 |
| Global fatigue score | 4.1 | 6.9 | 8.6 | 6.5 |
| **PROMIS - Fatigue Short Form 13a** | **Patient 1** | **Patient 2** | **Patient 3** | **Average Value (n=3)** |
| Raw scores | 31 | 36 | 51 | 39.3 |
| t-score | 55.3 | 58.2 | 66.5 | 60.0 |
| Standard deviation* |  |  |  | 1.0 |
| **Quality of Life** | | | | |
| **SF - 36** | **Patient 1** | **Patient 2** | **Patient 3** | **Average value (n=3)** |
| Physical functioning | 65 | 0 | 5 | 23.3 |
| Role functioning/ physical | 75 | 0 | 0 | 25.0 |
| Role functioning/ emotional | 66.7 | 0 | 100 | 55.6 |
| Energy/ fatigue | 30 | 20 | 10 | 20.0 |
| Emotional well-being | 60 | 44 | 64 | 56.0 |
| Social functioning | 62.5 | 12.5 | 0 | 25.0 |
| Pain | 55 | 57.5 | 10 | 40.8 |
| General Health | 30 | 20 | 15 | 21.7 |
| **EQ-5D-5L** | **Patient 1** | **Patient 2** | **Patient 3** |  |
| Health states | 32233 | 54532 | 42442 |  |
| Utility index | 0.5 | 0.048 | 0.044 |  |
| Standard deviation* | 1.7 | 3.9 | 3.9 |  |

# References

Amtmann, D., Cook, K.F., Jensen, M.P., Chen, W.-H., Choi, S., Revicki, D., et al. (2010). Development of A Promis Item Bank to Measure Pain Interference. *Pain* 150(1)**,** 173-182. doi: 10.1016/j.pain.2010.04.025.

Attal, N., Poindessous-Jazat, F., De Chauvigny, E., Quesada, C., Mhalla, A., Ayache, S.S., et al. (2021). Repetitive transcranial magnetic stimulation for neuropathic pain: a randomized multicentre sham-controlled trial. *Brain: A Journal of Neurology* 144(11)**,** 3328-3339. doi: 10.1093/brain/awab208.

Cella, D., Lai, J.-S., Jensen, S.E., Christodoulou, C., Junghaenel, D.U., Reeve, B.B., et al. (2016). Clinical Validity of the PROMIS® Fatigue Item Bank across Diverse Clinical Samples. *Journal of clinical epidemiology* 73**,** 128-134. doi: 10.1016/j.jclinepi.2015.08.037.

Cella, D., Riley, W., Stone, A., Rothrock, N., Reeve, B., Yount, S., et al. (2010). The Patient-Reported Outcomes Measurement Information System (PROMIS) developed and tested its first wave of adult self-reported health outcome item banks: 2005-2008. *Journal of Clinical Epidemiology* 63(11)**,** 1179-1194. doi: 10.1016/j.jclinepi.2010.04.011.

Cleeland, C.S. (2009). *The Brief Pain Inventory User Guide.*

Cleeland, C.S., and Ryan, K.M. (1994). Pain assessment: global use of the Brief Pain Inventory. *Annals of the Academy of Medicine, Singapore* 23(2)**,** 129-138.

Deandrea, S., Montanari, M., Moja, L., and Apolone, G. (2008). Prevalence of undertreatment in cancer pain. A review of published literature. *Annals of Oncology* 19(12)**,** 1985-1991. doi: 10.1093/annonc/mdn419.

Devlin, N., Brazier, J., Pickard, A.S., and Stolk, E. (2018). 3L, 5L, What the L? A NICE Conundrum. *PharmacoEconomics* 36(6)**,** 637-640. doi: 10.1007/s40273-018-0622-9.

EQ-5D (2019). *EQ-5D-5L User Guide.* EuroQol Research Foundation.

Ghajarzadeh, M., Jalilian, R., Sahraian, M.A., Moghadasi, A.N., Azimi, A., Mohammadifar, M., et al. (2018). Pain in Patients with Multiple Sclerosis. *Mædica* 13(2)**,** 125-130. doi: 10.26574/maedica.2018.13.2.125.

Güngör, D., Schober, A.K., Kruijshaar, M.E., Plug, I., Karabul, N., Deschauer, M., et al. (2013). Pain in adult patients with Pompe disease. *Molecular Genetics and Metabolism* 109(4)**,** 371-376. doi: 10.1016/j.ymgme.2013.05.021.

Hays, R.D., Sherbourne, C.D., and Mazel, R.M. (1993). The RAND 36-Item Health Survey 1.0. *Health Economics* 2(3)**,** 217-227. doi: 10.1002/hec.4730020305.

HealthMeasures *PROMIS® Score Cut Points* [Online]. Available: <https://www.healthmeasures.net/score-and-interpret/interpret-scores/promis/promis-score-cut-points> [Accessed May 11 2023].

Hubig, L., Sussex, A.-K., MacCulloch, A., Hughes, D., Graham, R., Morris, L., et al. Quality of Life with Late-Onset Pompe Disease: Qualitative Interviews and General Public Utility Estimation in the United Kingdom. *Journal of Health Economics and Outcomes Research* 10(1)**,** 41-50. doi: 10.36469/001c.68157.

Janssen, M.F., Bonsel, G.J., and Luo, N. (2018). Is EQ-5D-5L Better Than EQ-5D-3L? A Head-to-Head Comparison of Descriptive Systems and Value Sets from Seven Countries. *PharmacoEconomics* 36(6)**,** 675-697. doi: 10.1007/s40273-018-0623-8.

Janssen, M.F., Pickard, A.S., Golicki, D., Gudex, C., Niewada, M., Scalone, L., et al. (2013). Measurement properties of the EQ-5D-5L compared to the EQ-5D-3L across eight patient groups: a multi-country study. *Quality of Life Research* 22(7)**,** 1717-1727. doi: 10.1007/s11136-012-0322-4.

Jiang, R., Janssen, M.F.B., and Pickard, A.S. (2021). US population norms for the EQ-5D-5L and comparison of norms from face-to-face and online samples. *Qual Life Res* 30(3)**,** 803-816. doi: 10.1007/s11136-020-02650-y.

Kanters, T.A., Hagemans, M.L.C., van der Beek, N.A.M.E., Rutten, F.F.H., van der Ploeg, A.T., and Hakkaart, L. (2011). Burden of illness of Pompe disease in patients only receiving supportive care. *Journal of Inherited Metabolic Disease* 34(5)**,** 1045-1052. doi: 10.1007/s10545-011-9320-x.

Kruger, E., Aggio, D., de Freitas, H., and Lloyd, A. (2023). Estimation of Health Utility Scores for Glycogen Storage Disease Type Ia. *PharmacoEconomics - Open*. doi: 10.1007/s41669-023-00397-z.

Lai, J.-S., Cella, D., Choi, S., Junghaenel, D.U., Christodoulou, C., Gershon, R., et al. (2011). How Item Banks and Their Application Can Influence Measurement Practice in Rehabilitation Medicine: A PROMIS Fatigue Item Bank Example. *Archives of Physical Medicine and Rehabilitation* 92(10, Supplement)**,** S20-S27. doi: 10.1016/j.apmr.2010.08.033.

Li, K.K., Harris, K., Hadi, S., and Chow, E. (2007). What Should be the Optimal Cut Points for Mild, Moderate, and Severe Pain? *Journal of Palliative Medicine* 10(6)**,** 1338-1346. doi: 10.1089/jpm.2007.0087.

Liu, D., Weng, J.-S., Ke, X., Wu, X.-Y., and Huang, S.-T. (2022). The relationship between cancer-related fatigue, quality of life and pain among cancer patients. *International Journal of Nursing Sciences* 10(1)**,** 111-116. doi: 10.1016/j.ijnss.2022.12.006.

Mawla, I., Ichesco, E., Zöllner, H.J., Edden, R.A.E., Chenevert, T., Buchtel, H., et al. (2021). Greater Somatosensory Afference With Acupuncture Increases Primary Somatosensory Connectivity and Alleviates Fibromyalgia Pain via Insular γ-Aminobutyric Acid: A Randomized Neuroimaging Trial. *Arthritis & Rheumatology (Hoboken, N.J.)* 73(7)**,** 1318-1328. doi: 10.1002/art.41620.

Mendoza, T.R., Wang, X.S., Cleeland, C.S., Morrissey, M., Johnson, B.A., Wendt, J.K., et al. (1999). The rapid assessment of fatigue severity in cancer patients. *Cancer* 85(5)**,** 1186-1196. doi: 10.1002/(SICI)1097-0142(19990301)85:5<1186::AID-CNCR24>3.0.CO;2-N.

Pickard, A.S., Law, E.H., Jiang, R., Pullenayegum, E., Shaw, J.W., Xie, F., et al. (2019). United States Valuation of EQ-5D-5L Health States Using an International Protocol. *Value Health* 22(8)**,** 931-941. doi: 10.1016/j.jval.2019.02.009.

RAND Corporation *36-Item Short Form Survey (SF-36) Scoring Instructions* [Online]. Available: <https://www.rand.org/health-care/surveys_tools/mos/36-item-short-form/scoring.html> [Accessed April 29 2023].

Ritchie, E.K., Cella, D., Fabbiano, F., Pigneux, A., Kanda, Y., Ivanescu, C., et al. (2023). Patient-reported outcomes from the phase 3 ADMIRAL trial in patients with FLT3-mutated relapsed/refractory AML. *Leukemia & Lymphoma***,** 1-13. doi: 10.1080/10428194.2023.2186731.

Rothrock, N.E., Hays, R.D., Spritzer, K., Yount, S.E., Riley, W., and Cella, D. (2010). Relative to the General US Population, Chronic Diseases are Associated with Poorer Health-Related Quality of Life as Measured by the Patient-Reported Outcomes Measurement Information System (PROMIS). *Journal of Clinical Epidemiology* 63(11)**,** 1195-1204. doi: 10.1016/j.jclinepi.2010.04.012.

Serlin, R.C., Mendoza, T.R., Nakamura, Y., Edwards, K.R., and Cleeland, C.S. (1995). When is cancer pain mild, moderate or severe? Grading pain severity by its interference with function. *Pain* 61(2)**,** 277-284. doi: 10.1016/0304-3959(94)00178-H.

Shahid, A., Wilkinson, K., Marcu, S., and Shapiro, C. (2012). *STOP, THAT, and One Hundred Other Sleep Scales.* Springer.

Tan, G., Jensen, M.P., Thornby, J.I., and Shanti, B.F. (2004). Validation of the brief pain inventory for chronic nonmalignant pain. *The Journal of Pain* 5(2)**,** 133-137. doi: 10.1016/j.jpain.2003.12.005.

VanderZee, K.I., Sanderman, R., Heyink, J.W., and de Haes, H. (1996). Psychometric Qualities of the RAND 36-Item Health Survey 1.0: A Multidimensional Measure of General Health Status. *International Journal of Behavioral Medicine* 3(2)**,** 104. doi: 10.1207/s15327558ijbm0302_2.

Ware, J.E., and Sherbourne, C.D. (1992). The MOS 36-item short-form health survey (SF-36). I. Conceptual framework and item selection. *Medical Care* 30(6)**,** 473-483.
